# Antimalarial and antiplasmodial activity of husk extract and fractions of *Zea mays*

**DOI:** 10.1080/13880209.2017.1302966

**Published:** 2017-03-21

**Authors:** Jude E. Okokon, Bassey S. Antia, Dinesh Mohanakrishnan, Dinkar Sahal

**Affiliations:** a Department of Pharmacology and Toxicology, Faculty of Pharmacy, University of Uyo, Uyo, Nigeria;; b Malaria Research Laboratory, International Centre for Genetic Engineering and Biotechnology, New Delhi, India;; c Department of Chemistry, University of Uyo, Uyo, Nigeria

**Keywords:** Herbal medicine, ethnopharmacology, *Plasmodium berghei*, plasmodium falciparum, tropical diseases

## Abstract

**Context:***Zea mays* L. (Poacae) husk decoctions are traditionally used in the treatment of malaria by various tribes in Nigeria.

**Objective:** To assess the antimalarial and antiplasmodial potentials of the husk extract and fractions on malaria parasites using *in vivo* and *in vitro* models.

**Materials and methods:** The ethanol husk extract and fractions (187–748 mg/kg, p.o.) of *Zea mays* were investigated for antimalarial activity against *Plasmodium berghei* using rodent (mice) malaria models and *in vitro* activity against chloroquine sensitive (Pf 3D7) and resistant (Pf INDO) strains of *Plasmodium falciparum* using the SRBR green assay method. Median lethal dose and cytotoxic activities against HeLa and HEKS cells were also carried out. The GCMS analysis of the most active fraction was carried out.

**Results:** The husk extract (187–748 mg/kg, p.o.) with LD_50_ of 1874.83 mg/kg was found to exert significant (*p* < 0.05–0.001) antimalarial activity against *P. berghei* infection in suppressive, prophylactive and curative tests. The crude extract and fractions also exerted prominent activity against both chloroquine sensitive (Pf 3D7) and resistant (Pf INDO) strains of *P. falciparum* with the ethyl acetate fraction exerting the highest activity with IC_50_ values of 9.31 ± 0.46 μg/mL (Pf 3D7) and 3.69 ± 0.66 μg/mL (Pf INDO). The crude extract and fractions were not cytotoxic to the two cell lines tested with IC_50_ values of >100 μg/mL against both HeLa and HEKS cell lines.

**Discussion and conclusion:** These results suggest that the husk extract/fractions of *Zea mays* possesses antimalarial and antiplasmodial activities and these justify its use in ethnomedicine to treat malaria infections.

## Introduction

*Zea mays* L. (Poacae), known as maize or corn, is an annual grass plant cultivated for human consumption and as animal feed. It was introduced to Nigeria in the sixteenth century (Osagie & Eka 1998). It is tall with strong erect stalks and a fibrous root system. The plant has long narrow leaves that are spaced alternately on opposite side of the stem and bears ears that are enclosed in modified leaves known as husks (Simmonds 1979). Besides its nutritive values, maize grains, leaves, cornsilks, stalk, and inflorescence are also used in ethnomedicine for the treatment of several ailments. The corn silk is used as an antidiabetic or diuretic, and decoction of the silk is consumed for the treatment of urinary troubles and gallstones (Foster & Duke 1990; Gill 1992; Abo et al. 2008). The ash of the cob is used for the treatment of cough (Gill 1992) and inflammatory diseases. The husks are used for the treatment of pains and arthritis (Owoyele et al. 2010). Warm tea of the husks is used for the treatment of malaria and diabetes in Ibibio traditional medicine. Biological activities reported on the husk extract include; analgesic, anti-inflammatory (Owoyele et al. 2010), and antioxidant (Dong et al. 2014) activities. Arabinoxylan, which has immunological effects, has been isolated from the husk extract (Ogawa et al. 2005), while eight phenolic compounds (gallic acid, protocatechuic acid, chlorogenic acid, cafeic acid, femlic acid, rutin, resveratrol, and kaempferol) have also been detected in ethanol husk extract of *Zea mays* (Dong et al. 2014). Information on the biological activities of the husk extract is scarce. We report in this study the antimalarial and antiplasmodial activity of the husk extract and fractions to confirm its use as malarial remedy in Ibibio ethnomedicine.

## Materials and methods

### Collection of plant materials

Fresh husks of *Zea mays* were collected in August, 2015 from Farmland in Uyo in Uyo LGA, Akwa Ibom State, Nigeria. The husks were identified and authenticated as *Zea mays* by Dr. Margaret Bassey, a taxonomist in the Department of Botany and Ecological studies, University of Uyo, Uyo, Nigeria. Herbarium specimen (FPH, 614) was deposited at the Faculty of Pharmacy Herbarium, University of Uyo, Uyo.

### Extraction

The plant parts (husks) were washed, cut into smaller pieces and air-dried for 2 weeks. The dried husks were pulverized using a pestle and mortar. The powdered husk was macerated in 50% ethanol for 72 h. The liquid ethanol extract obtained by filtration was evaporated to dryness in a rotary evaporator 40 °C. The crude ethanol husk extract (20 g) was dissolved in 200 mL of distilled water and further partitioned successively into each of petroleum ether, chloroform, ethyl acetate and *n*-butanol to give the corresponding fractions of these solvents, while the residue was taken as aqueous fraction. The extract (yield 2.83%) and fractions were stored in a refrigerator at −4 °C until they were used for the experiments reported in this study.

### Phytochemical screening

Phytochemical screening of the crude husk extract was carried out employing standard procedures and tests (Trease & Evans 1989; Sofowora 1993), to reveal the presence of chemical constituents such as alkaloids, flavonoids, tannins, terpenes, saponins, anthraquinones, reducing sugars and cardiac glycosides.

### Animals

Thirty Swiss albino mice (18–25 g) of either sex divided into five groups of 6 mice each per model were used for these experiments. The animals were housed in standard cages and were maintained on a standard pelleted feed (Guinea feed) and water *ad libitum*. Permission and approval for animal studies were obtained from the College of Health Sciences Animal Ethics Committee, University of Uyo.

### Parasites

A chloroquine sensitive strain of *Plasmodium berghei* (ANKA) was obtained from the National Institute of Medical Research (NIMER), Yaba Lagos, Nigeria and was maintained by sub-passage in mice. While *Plasmodium falciparum* strains *Pf* 3D7 and *Pf* INDO were obtained from the International Center for Genetic Engineering and Biotechnology, New Delhi, India.

### Determination of acute toxicity of crude husk extract

The median lethal dose (LD_50_) was determined for estimating acute toxicity of the crude husk extract in Swiss albino mice model using the method of Lorke (1983). This involved intraperitoneal administration of different doses of the extract (1000–5000 mg/kg) to groups of three mice each. The animals were observed for manifestation of physical signs of toxicity such as writhing, decreased motor activity, decreased body/limb tone, decreased respiration and death. The number of deaths in each group within 24 h was recorded.

### Parasite inoculation

Each mouse used in the experiment was inoculated intraperitoneally with 0.2 mL of infected blood containing about 1 × 10^7^ *P. berghei berghei* parasitized erythrocytes. The inoculum consisted of 5 × 10^7^ *P. berghei berghei* erythrocytes per mL. This was prepared by determining both the percentage parasitaemia and the erythrocytes count of the donor mouse and diluting the blood with isotonic saline in proportions indicated by both determinations (Odetola & Basir 1980).

### Drug administration

The drug (artesunate) and extract used in the *in vivo* antiplasmodial study were orally administered with the aid of a stainless metallic feeding cannula.

### Evaluation of *in vivo* anti-malarial activity of ethanol crude extract

#### Evaluation of suppressive activity of the extract (4-day test)

This test was used to evaluate the schizontocidal activity of the extract and artesunate against early *P. berghei berghei* infection in mice. This was done as described by Knight and Peters (1980). Thirty mice were randomly divided into five groups of six mice each. On the first day (D_0_), the 30 mice were infected with the parasite and randomly divided into various groups. These were orally administered with the extract and artesunate. The mice in group 1 were administered 187 mg/kg, group 2, 374 mg/kg and group 3, 748 mg/kg of crude husk extract, while group 4 was administered 5 mg/kg of artesunate (positive control), and 10 mL/kg of distilled water was administered to group 5 (negative control) for four consecutive days (D_0_–D_3_) between 8 am and 9 am. On the fifth day (D_4_), thin blood film was made from tail blood of each mouse. The film was then stained with Giemsa stain to reveal parasitized erythocytes out of 500 in a random field of the microscope. The average percentage suppression of parasitaemia was calculated in comparison with the controls as follows:

$$\frac{\text{Average }\%\text{ parasitaemia in negative control}\;-\text{Average \% parasitaemiain Positive groups }}{\text{Average \% parasitaemia in negative control}}\times 100$$

#### Evaluation of prophylactic or repository activities of extract

The repository activity of the extract and artesunate was assessed by using the method described by Peters (1965). The mice were randomly divided into five groups of six mice each. Groups 1–3 were orally administered 187, 374 and 748 mg/kg/day of the husk extract respectively, Groups 4 and 5 were respectively administered 5 mg/kg/day of artesunate (positive control) and 10 mL/kg of distilled water (negative control). Administration of the extract/drug continued for three consecutive days (D_0_–D_2_). On the fourth day (D_3_), the mice were inoculated with *P. berghei berghei.* The parasitaemia level of each mouse was assessed by blood smear 72 h later.

#### Evaluation of curative activities of extract (Rane’s test)

This was used to evaluate the schizontocidal activity of the extract, and artesunate in established infection. This was done as described by Ryley and Peters (1970). *Plasmodium berghei berghei* was injected intraperitoneally into another 30 mice on the first day (D_0_). Seventy-two hours later (D_3_), the mice were divided randomly into five groups of six mice each. Different doses of the extract, 187, 374 and 748 mg/kg were orally administered, respectively, to mice in groups 1–3, 5 mg/kg/day of artesunate was administered to the group 4 (positive control) and group 5 was given 10 mL/kg of distilled water (negative control). The extract and drugs were administered once daily for 5 days. Giemsa stained thin smears were prepared from tail blood samples collected on each day of treatment to monitor parasitaemia level. The mean survival time (MST) of the mice in each treatment group was determined over a period of 29 days (D_0_–D_28_).

$$\frac{\text{No of days survived}}{\text{Total No}.\text{ of days }\left(\text{29}\right)}\times 100=\text{MST}$$

### Evaluation of *in vitro* antiplasmodial activity

#### *In vitro* cultivation of *Plasmodium falciparum*

CQ-sensitive strain 3D7 and CQ-resistant strain INDO of *P. falciparum* were used in this study to assess the antiplasmodial activity of the crude husk extract and fractions on erythrocytic stages *in vitro*. The culture was maintained at the Malaria Research Laboratory, International Centre for Genetic Engineering and Biotechnology, New Delhi, India. *Plasmodium falciparum* culture was maintained according to the method described by Trager and Jensen (1976) with minor modifications. *Plasmodium falciparum* (3D7) cultures were maintained in fresh O +ve human erythrocytes suspended at 4% hematocrit in RPMI 1640 (Sigma) containing 0.2% sodium bicarbonate, 0.5% albumax, 45 μg/L hypoxanthine, and 50 μg/L gentamicin and incubated at 37 °C under a gas mixture of 5% O_2_, 5% CO_2_, and 90% N_2_. Every day, infected erythrocytes were transferred into fresh complete medium to propagate the culture. For *Plasmodium falciparum* (INDO strain) in culture medium, albumax was replaced by 10% pooled human serum.

#### Drug dilutions

Dimethyl sulfoxide (DMSO) was used to prepare the stock solutions of husk extract, fractions and artemisinin, while water (Milli-Q grade) was used in the case of CQ stock solution. Culture medium was used to dilute the stock solutions to their required concentrations exception of CQ. The final solution of each stock was constituted to contained nontoxic concentration of DMSO (0.4%), which was found to be harmless to the parasite. Drugs, husk extract and fractions were then placed in 96-well flat bottom tissue culture grade plates.

#### *In vitro* antiplasmodial assays

The crude husk extract and fractions of this plant were evaluated for their antiplasmodial activity against 3D7 and INDO strains of *Plasmodium falciparum*. For drug screening, SYBR green I-based fluorescence assay was set up as described previously (Smilkstein et al. 2004). Sorbitol synchronized parasites were incubated under normal culture conditions at 2% hematocrit and 1% parasitemia in the absence or presence of increasing concentrations of husk extract and fractions. CQ and artemisinin were used as positive controls, while 0.4% DMSO was used as the negative control. After 48 h of incubation, 100 μL of SYBR Green I solution (0.2 μL of 10,000 × SYBR Green I (Invitrogen)/mL) in lysis buffer {Tris (20 mM; pH 7.5), EDTA (5 mM), saponin (0.008%, w/v), and Triton X-100 (0.08%, v/v)} was added to each well and mixed twice gently with multi-channel pipette and incubated in dark at 37 °C for 1 h. Fluorescence was measured with a Victor fluorescence multi-well plate reader (Perkin Elmer) with excitation and emission wavelength bands cantered at 485 and 530 nm, respectively. The fluorescence counts were plotted against the drug concentration and the 50% inhibitory concentration (IC_50_) was determined by analysis of dose–response curves and IC_50_ estimator. Results were validated microscopically by examination of Giemsa-stained smears of extract/fraction-treated parasite cultures.

#### Cytotoxic activity on HeLa and HEKS cells using MTT assay

The cytotoxic effects of extract and fractions on host cells were assessed by functional assay as described previously (Mosmann 1983) using HeLa cells cultured in RPMI containing 10% foetal bovine serum, 0.21% sodium bicarbonate (Sigma) and 50 μg/mL gentamicin (complete medium) and human embroynic kidney 293 cells cultured in DMEM and supplemented with 10% foetal bovine albumin. Briefly, cells (10^4^ cells/200 μL/well) were seeded into 96-well flat-bottom tissue culture plates in complete medium. Drug solutions were added after 24 h of seeding and incubated for 48 h in a humidified atmosphere at 37 °C and 5% CO_2_. DMSO (as positive inhibitor) was added at 10%. A stock solution (20 μL) of MTT (5 mg/mL in 1 × phosphate buffered saline) was added to each well, gently mixed and incubated for another 4 h. After spinning the plate at 1500 rpm for 5 min, supernatant was removed and 100 μL of DMSO (stop agent) was added. Formation of formazon was read on a microtiter plate reader (Versa max tunable multi-well plate reader) at 570 nm. The 50% cytotoxic concentration (TC_50_) of drug was determined by analysis of dose–response curves and IC_50_ estimator.

### Gas chromatography-mass spectrometry analysis

Quantitative and qualitative data were determined by GC and GC-MS, respectively. The fraction was injected onto a Shimadzu GC-17 A system, equipped with an AOC-20i autosampler and a split/splitless injector. The column used was an DB-5 (Optima-5), 30 m, 0.25 mm i.d., 0.25 μm df, coated with 5% diphenyl-95% polydimethylsiloxane, operated with the following oven temperature program: 50 °C, held for 1 min, rising at 3 °C/min to 250 °C, held for 5 min, rising at 2 °C/min to 280 °C, held for 3 min; injection temperature and volume, 250 °C and 1.0 μL, respectively; injection mode, split; split ratio, 30:1; carrier gas, nitrogen at 30 cm/s linear velocity and inlet pressure 99.8 KPa; detector temperature, 280 °C; hydrogen, flow rate, 50 mL/min; air flow rate, 400 mL/min; make-up (H_2_/air), flow rate, 50 mL/min; sampling rate, 40 ms. Data were acquired by means of GC solution software (Shimadzu). Agilent 6890 N GC was interfaced with a VG Analytical 70–250 s double-focusing mass spectrometer. Helium was used as the carrier gas. The MS operating conditions were: ionization voltage 70 eV, ion source 250 °C. The GC was fitted with a 30 m × 0.32 mm fused capillary silica column coated with DB-5. The GC operating parameters were identical with those of GC analysis described above.

### Identification of the compounds

The identification of components present in the active fraction of the plants’ extract was based on direct comparison of the retention times and mass spectral data with those for standard compounds, and by computer matching with the Wiley and Nist Libraries (Adams 2001; Setzer et al. 2007).

### Statistical analysis and data evaluation

Data obtained from this work were analyzed statistically using Student’s *t*-test and ANOVA (One-way) followed by a post test (Turkey–Kramer multiple comparison test). Differences between means was considered significant at 1% and 5% level of significance, that is *p* ≤ 0.01 and 0.05.

## Results

### Phytochemical screening

Results of phytochemical screening of the crude ethanol husk extract revealed the presence of chemical constituents such as alkaloids, flavonoids, tannins, terpenes, saponins, cardiac glycosides and sugars.

### Determination of acute toxicity of crude husk extract

The median lethal dose (LD_50_) of the crude husk extract was calculated to be 1874.83 mg/kg. The physical signs of toxicity observed included excitation, paw licking, increased respiratory rate, decreased motor activity, gasping and coma, followed by death.

### Effect on suppressive activity of ethanol husk extract of *Zea mays*

The extract showed a dose-dependent chemosuppressive effect on the parasitaemia. These effects were statistically significant relative to the control (*p* < 0.05–0.001). The chemoinhibitory percentages ranged from 34.58 to 69.18 (Table 1). However, the effect of the extract was weak compared to that of the standard drug, artesunate, with a chemosuppression of 98.82% (Table 1).

**Table 1.** *In vivo* suppressive activity of crude ethanol husk extract of *Zea mays* (4-day test) against *P. berghei* infection in mice.

Drug/extract	Dose (mg/kg)	Parasitaemia	% Chemosuppression
Distilled water extract	10 mL/kg	44.33 ± 2.18	–
	187	29.00 ± 1.02^a^	34.58
	374	20.66 ± 0.33^a^	53.39
	748	13.66 ± 1.45^a^	69.18
Artesunate	5	0.52 ± 0.01^a^	98.82

Values are expressed as mean ± S.E.M. Significance relative to control.

a *p* < 0.001: *n* = 6.

### Effect on repository activity of ethanol husk extract of *Zea mays*

The ethanol husk extract of *Zea mays* showed a dose-dependent chemosuppressive effect (65.89–81.85%) on the parasitaemia during prophylactic studies. These effects were statistically significant relative to the control (*p* < 0.001), but weak compared to that of the standard drug, artesunate, with chemosuppression of 90.92% (Table 2).

**Table 2.** Repository/Prophylactic activity of ethanol husk extract of *Zea mays* against *Plasmodium berghei* infection in mice.

Treatments	Dose(mg/kg)	Parasitaemia	% Chemosuppression
Normal saline	10 mL/kg	14.66 ± 0.66	–
Crude extract	187	5.00 ± 3.24^a^	65.89
	374	4.66 ± 0.90^a^	68.21
	748	2.66 ± 1.33^a^	81.85
Artesunate	5.0	1.33 ± 0.66^a^	90.92

Values are expressed as Mean ± SEM. Significance relative to control.

a *p* < 0.001, *n* = 6.

### Antiplasmodial effect of ethanol husk extract of *Zea mays* on established infection

**Figure 1.**
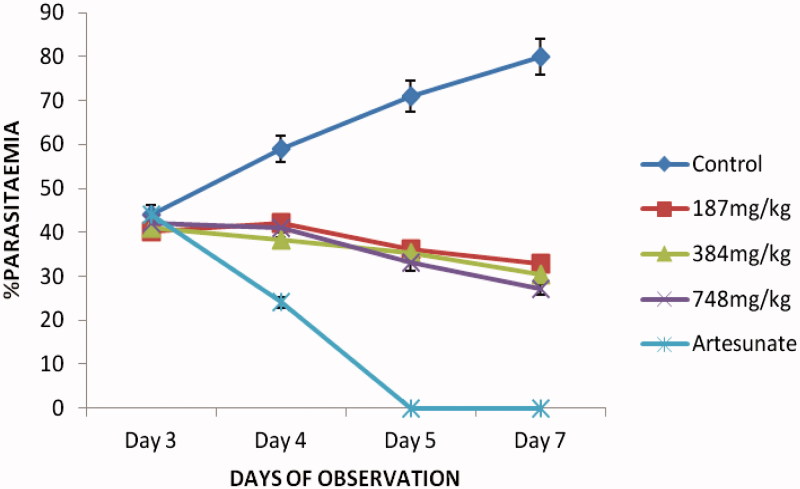
*In vivo* curative antiplasmodial activity of crude ethanol husk extract of *Zea mays* (Rane’s test) against *P. berghei* infection in mice.

The extract showed a dose-dependent schizonticidal effect on the parasitaemia. There were reductions in the percentage parasitaemia of the extract/artesunate-treated groups compared to that of the control in which prominent increases were recorded. These reductions were statistically significant relative to the control (*p* < 0.05–0.001) (Figure 1). Though the extract showed a significant (*p* < 0.05–0.001) dose-dependent mean survival time on established infection, the effect of the extract (187–748 mg/kg) was weak compared to that of the standard drug, artesunate (Table 3).

**Table 3.** Mean Survival Time (MST) of mice receiving different doses of ethanol husk extract of *Zea mays* during established infection.

Drug/extract	Dose (mg/kg)	MST (days)
Distilled water extract	10 mL/kg	14.33 ± 0.33
	187	15.33 ± 0.66
	374	19.0 ± 1.00^a^
	748	24.00 ± 1.73^b^
Artesunate	5	30.00 ± 0.00^b^

Values are expressed as Mean ± S.E.M. Significance relative to control.

a *p* < 0.05.

b *p* < 0.001, *n* = 6.

### *In vitro* antiplasmodial and cytotoxic activities

The results of the *in vitro* studies showed that the plant extract and fractions displayed antiplasmodial activity against chloroquine sensitive Pf 3D7 and resistant Pf INDO strains of *P. falciparum* (Table 4). The ethyl acetate fraction was found to exhibit promising activity against both strains of *P. falciparum* with IC_50_ values of 9.31 ± 0.46 μg/mL (Pf 3D7) and 3.69 ± 0.66 μg/mL (Pf INDO). The potency order was ethyl acetate > chloroform > butanol > crude extract > petroleum ether. The crude extract and fractions were not cytotoxic to the two cell lines tested with TC_50_ of >100 μg/mL against both HeLa and HEKS cell lines.

**Table 4.** *In vitro* antiplasmodial activities of crude husk extract and fractions of *Z. mays*.

Crude extract/fraction	IC_50_(μg/mL) *Pf* 3D7	IC_50_(μg/mL) *Pf* INDO	Cytotoxicity	
			Hela cells TC_50_(μg/mL)	HEKS cells TC_50_(μg/mL)
Crude extract	45.52 ± 0.86	38.51 ± 0.18	>100	>100
Pet. ether	47.88 ± 0.36	49.11 ± 0.15	>100	>100
Chloroform	8.46 ± 0.37	3.84 ± 0.32	>100	>100
Ethyl acetate	9.31 ± 0.46	3.69 ± 0.66	>100	>100
Butanol	35.35 ± 0.16	44.81 ± 0.12	>100	25.0
Aqueous	>100	>100	>100	>100

Values are expressed as mean ± S.E.M.

### GCMS analysis

The GCMS analysis of the ethyl acetate fraction of *Zea mays* revealed the presence of bioactive compounds with major and minor ones as represented in Table 5.

**Table 5.** GCMS analysis of ethyl acetate fraction of *Zea mays* husk.

Peak	RT	Compound name	Formula	Mol. mass
1.	4.474	1,2,3-Propanetriol	C_3_H_8_O_3_	92
2.	8.875	2,3-Dihydro-3,5-dihydroxy-6-methyl-4h-pyran-4-one	C_6_H_8_O_4_	144
3.	10.088	Butanedioic acid, hydroxy-, diethyl ester, (.+/–.)-	C_8_H_14_O_5_	190
4.	11.213	2,3-Dihydro-benzofuran	C_8_H_8_O	120
5.	12.397	2H-Pyran-2-one, tetrahydro-4-hydroxy-4-methyl-	C_6_H_10_O_3_	130
6.	13.261	2-Methoxy-4-vinylphenol	C_9_H_10_O_2_	150
7.	14.920	1-Tridecene	C_13_H_26_	182
8.	16.315	Ethyl .beta.-d-riboside	C_7_H_14_O	178
9.	17.526	1*R*-Ethoxy-3-trans-methoxy-2-cis-methylcyclohexane	C_10_H_20_O_2_	172
10.	18.092	Benzeneacetic acid, 4-hydroxy-, methyl ester	C_9_H_10_O_3_	166
11.	18.258	Dodecanoic acid, methyl ester	C_13_H_26_O_2_	214
12.	19.684	Dodecanoic acid	C_12_H_24_O_2_	200
13.	19.833	Trifluoroacetic acid,n-tridecyl ester	C_15_H_27_F_3_O_2_	296
14.	19.912	Dodecanoic acid, ethyl ester	C_14_H_28_O_2_	228
15.	22.757	Delta.1,.alpha.-Cyclohexaneacetic acid	C_8_H_12_O_2_	140
16.	23.820	4-((1*E*)-3-Hydroxy-1-propenyl)-2-methoxyphenol	C_10_H_12_O_3_	180
17.	24.027	Tetradecanoic acid	C_14_H_28_O_2_	228
18.	24.297	n-Tetracosanol-1	C_24_H_50_O	354
19.	24.351	Tetradecanoic acid, ethyl ester	C_16_H_32_O_2_	256
20.	25.912	p-Hydroxycinnamic acid, ethyl ester	C_11_H_12_O_3_	192
21.	27.149	Hexadecanoic acid, methyl ester	C_17_H_34_O_2_	270
22.	27.451	Ethyl (2*E*)-3-(4-hydroxy-3- methoxyphenyl)-2-propenoate	C_12_H_14_O_4_	222
23.	28.537	Pentadecanoic acid	C_15_H_30_O_2_	242
24.	28.879	Hexadecanoic acid, ethyl ester	C_18_H_36_O_2_	284
25.	31.944	Ethyl (9*Z*,12*Z*)-9,12- octadecadienoate	C_20_H_36_O_2_	308
26.	32.111	9-Octadecenoic acid, methyl ester	C_19_H_36_O_2_	296
27.	32.756	Ethyl (9*Z*,12*Z*)-9,12-octadecadienoate	C_20_H_36_O_2_	308
28.	33.553	9-octadecenoic acid (*Z*)-, ethyl ester	C_19_H_36_O_2_	296
29.	33.664	Octadecanoic acid, methyl ester	C_19_H_38_O_2_	298
30.	34.161	Nonadecanoic acid, ethyl ester	C_21_H_42_O_2_	326
31.	36.833	2-Hydroxy-3-[(9*E*)-9-octadecenoyloxy]propyl (9*E*)-9-octadecenoate	C_39_H_72_O_5_	620
32.	37.072	1,2-Benzenedicarboxylic acid	C_24_H_38_O_4_	390
33.	38.028	Docosanoic acid, ethyl ester	C_24_H_48_O_2_	368
34.	38.784	1,1'-Biphenyl-3,4,4'-trimethoxy-6'-formyl-	C_16_H_16_O_4_	272
35.	39.157	(*R*-(R*,R*))-4-(1,5-Dimethylhexyl)-1-cyclohexenecarboxylic acid	C_15_H_26_O_2_	238
36.	41.091	Docosanoic acid, ethyl ester	C_24_H_48_O_2_	368
37.	42.365	Pentacosane, 1-bromo-	C_25_H_51_Br	430
38.	46.599	Stigmast-5-en-3-ol, (3.Beta.)-	C_29_H_50_O	414
39.	49.936	Ergost-5-en-3-ol, (3.Beta.,24*R*)-	C_28_H_48_O	400
40.	50.813	Stigmasterol	C_29_H_48_O	412
41.	52.610	Stigmast-5-en-3-ol, (3.Beta.)-	C_29_H_50_O	414

## Discussion

The husk extract of *Zea mays* used as malarial remedy by the Ibibio tribe of Nigeria was investigated for *in vivo* and *in vitro* antiplasmodial activities using standard models. Acute toxicity and cytotoxicity studies as well as phytochemical studies of the husk extract and fractions were carried out.

The median lethal dose (LD_50_) which was determined to be 1874.83 mg/kg was found to be relatively safe with insignificant toxicity (Homburger 1989). The results of the *in vivo* study revealed that the crude extract significantly reduced parasitaemia in prophylactic, suppressive and curative models in a dose-dependent fashion confirming the antimalarial potential of this extract. These findings were further supported by the results of the mean survival time of the extract-treated mice which was significantly prolonged compared to those of the control group demonstrating a significant protection potential of the extract. The results of *in vitro* study revealed that the husk extract and fractions had antiplasmodial activity against both chloroquine sensitive (3D7) and resistant (INDO) strains of *Plasmodium falciparum* with ethyl acetate fraction as the most active fraction. This suggests the localization of the active molecules in the ethyl acetate fraction. The results of the *in vitro* activity corroborated that of the *in vivo* study and confirm the antimalarial and antiplasmodial potentials of the husk extract. These findings further lay credence to the use of the husk of *Zea mays* in the treatment of malaria traditionally. Besides, the fact that the extract and fractions were active against the chloroquine-resistant strain (INDO) of *P. falciparum* implies that the husk extract can be an effective agent against chloroquine resistant malaria. The activities of the crude husk extract and fractions are predicated on the chemical constituents of the extract and fractions as revealed by the results of phytochemical screening of the crude husk extract and GCMS analysis of the active fraction.

The husk extract of *Zea mays* was found in this study to contain alkaloids, saponins, tannins, phlabatannins, flavonoids and cardiac glycosides. Some secondary metabolites of plants such as alkaloids, flavonoids and triterpenoids have been reported to possess antiplasmodial activity (Kirby et al. 1989; Philipson & Wright 1991; Christensen & Kharazmi 2001). These chemical compounds which are present in this extract and fractions may in part be responsible for the observed antimalarial and antiplasmodial activities. Also, the GCMS analysis of the active ethyl acetate fraction revealed the presence of some pharmacologically active compounds such as phenolics and polyunsaturated fatty acids (PUFA) especially C-18 fatty acids among others. Polyunsaturated fatty acids such as *p*-hydroxycinnamic acid ethyl ester, stigmasterol, docosanoic acid ethyl ester, octadecanoic acid methyl ester, 9-octadecenoic acid (*Z*)-ethyl ester and hexadecanoic acid ethyl ester have been implicated in antiplasmodial activity and this activity has been reported to increase with the degree of unsaturation (Kumaratilake et al. 1992; Krugliak et al. 1995; Suksamrarn et al. 2005; Attioua et al. 2007; Melariri et al. 2011, 2012; Zofou et al. 2011, 2012; Zhai et al. 2014). Similarly, Dong et al. (2014) had reported the presence of polyphenolics such as gallic acid, protocatechuic acid, chlorogenic acid, cafeic acid, femlic acid, rutin, resveratrol, and kaempferol in the husk extract *Zea mays*. Gallic acid and kaempferol are implicated in the antiplasmodial activities of plants (Horgen et al. 1997; Teffo et al. 2010; Barliana et al. 2014). Rutin has been shown to possess significant antiplasmodial activity against chloroquine sensitive and resistant strains of *P. falciparum* with IC_50_ of 3.53 ± 13.34 μM against 3D7 and 15.00 μM against K1 (Attioua et al. 2011). These compounds present in this fraction are likely to be responsible for the observed antiplasmodial activity. Besides, antioxidant potentials of some plant and natural products especially flavonoids have been found to promote schizonticidal activity by modulating the cellular signalling pathway (Al-Adhroey et al. 2011) and this has been suggested to be responsible for antiplasmodial activity of compounds such as quercetin (Cimanga et al. 2009; Ganesh et al. 2012), as elevated free radicals levels which are common features of malaria disease are implicated in severe malaria complications. This could be one of the modes of action of this extract as it contains phenolics and flavonoids with antioxidant activity (Dong et al. 2014).

Flavonoids are known to exert antiplasmodial activity by chelating with nucleic acid base pairing of the parasite (Lui et al. 1992), while the plasmodicidal activities of terpenes and their derivatives such as monoterpenes and sesquiterpenes have been linked to endoperoxidation (Hatzakis et al. 2007).

These compounds (flavonoids and terpenes) present in this plant extract may have contributed to the plasmodicidal activity of this extract and therefore explained the mechanism of antiplasmodial effect of the extract.

## Conclusion

The results of this study indicate that the husk of *Zea mays* plant possesses significant *in vivo* antimalarial activity against *P. berghei* infection in mice and *in vitro* antiplasmodial activity against chloroquine sensitive and resistant strains of *P. falciparum.* These findings justify and confirm the ethno botanical usage of this plant in the treatment of malaria. Therefore, further research on ethanol husk extract and fractions of *Zea mays* could be carried out in order to isolate, identify and characterize the active principle from this plant.
